# Citric Acid in Rice Root Exudates Enhanced the Colonization and Plant Growth-Promoting Ability of Bacillus altitudinis LZP02

**DOI:** 10.1128/spectrum.01002-22

**Published:** 2022-10-20

**Authors:** Huiwen Jiao, Weihui Xu, Yunlong Hu, Renmao Tian, Zhigang Wang

**Affiliations:** a School of Life Science and Agriculture Forestry, Qiqihar University, Qiqihar, Heilongjiang, China; b Heilongjiang Provincial Technology Innovation Center of Agromicrobial Preparation Industrialization, Qiqihar, China; c Institute for Food Safety and Health, Illinois Institute of Technology, Chicago, Illinois, USA; University of Massachusetts Amherst

**Keywords:** promotes plant growth, chemotaxis, citric acid, interaction

## Abstract

Exploration of the underlying mechanisms of plant-microbe interactions is very important. In the present study, citric acid in the root exudates of rice significantly enhanced the colonization of Bacillus altitudinis LZP02 in the rhizosphere. According to the results of transcriptome and reverse transcription-quantitative PCR or analyses, citric acid increased the expression of several genes involved in bacterial chemotaxis and biofilm formation in B. altitudinis LZP02. In addition, citric acid also increased the expression of several genes associated with *S*-adenosylmethionine biosynthesis and metabolism. Interestingly, the secretion of citric acid by rice roots could be increased by inoculation with B. altitudinis LZP02. The result indicated that citric acid might be a vital signal in the interaction between rice and B. altitudinis LZP02. Further verification showed that citric acid enhanced the plant growth-promoting ability of B. altitudinis LZP02.

**IMPORTANCE** In a previous study, the mechanism by which citric acid in rice root exudates enhanced the colonization of Bacillus altitudinis LZP02 was discovered. The present study verified that citric acid increased the recruitment and rice growth-promoting ability of B. altitudinis LZP02. These findings serve as an interesting case for explaining the underlying mechanisms of plant-microbe interactions. Henceforth, citric acid and B. altitudinis LZP02 could be exploited for the development of sustainable agronomy.

## INTRODUCTION

Chemotaxis is the ability of motile soil bacteria to sense and adjust their movements along gradients of compounds and constitutes a positive role during rhizosphere colonization (1, 2). Plant colonization by plant growth-promoting rhizobacteria (PGPR) is a highly relevant process for the establishment of both green and sustainable agricultural development (3). The plant rhizosphere is a unique position for plant-microbe interactions. The bacteria density orientation in the rhizosphere is aided by root exudates (1). Chemotaxis toward root exudates activates rhizobacterial recruitment and the establishment of bacterium-root interactions. Root exudates promote the growth of soil PGPR and support biofilm formation by microbes (4). Studies have shown that, compared with bacterial strains in which the biofilm is sufficient, the colonization of biofilm-deficient strains was much lower (5). The cell motility relies on the rotation of the flagellum of bacteria; thus, enhancement of flagellar assembly may lead to successful root colonization (6). Organic acids and amino acids are the main components of primary metabolites in root exudates and are secreted and passively lost from roots (7). A previous study has shown that chemotactic responses of Bacillus velezensis B26 were induced by organic acids and citric acids enhanced the gene expression connected to the biofilm (8). Another study demonstrated that Escherichia coli and Bacillus subtilis movement toward amino acids can be utilized quickly (9). Malic acid, citric acid, and succinic acid have been shown to significantly promote B. amyloliquefaciens T-5 recruitment, enhancing the rhizobacteria population (10). Moreover, Bacillus subtilis RR4 can move toward malic acid released from rice roots (11).

The bacterial chemotaxis pathway of rhizospheric *Bacillus* includes methyl-accepting chemotaxis proteins (MCPs) that sense signals from root exudates (12). This interaction produces a stimulus that modulates CheA kinase activity (13, 14). The effector protein CheY of the two-component system is rapidly phosphorylated to interact with the flagellar motor and causes changes in the direction of bacterial movement (15). In *pseudosolanacearum* Ps29, McpC and McpP mediate the chemotaxis response toward citrate (16). In Bacillus subtilis, McpA was the major chemoreceptor response to phenol (17). However, in Bacillus velezensis, McpA is a principal chemoreceptor for the chemotactic response to d-galactose in cucumber root exudates (18). The interactions between root exudates and MCPs are crucial for bacterial motility and colonization of the plant root (13). However, the MCPs that mediate chemotaxis toward root exudates differ across bacterial species, and the ligands of the homologous MCPs vary across different bacteria (19).

Bacillus altitudinis LZP02 is an efficient plant growth-promoting rhizobacterium that interacts closely with plant roots. In a previous study, we comprehensively identified the chemoattractants of B. altitudinis LZP02 in rice root exudates. Compared with the different kinds and concentrations of chemoattractants, 100 μmol L^−1^ citric acid significantly enhanced the motility and colonization of B. altitudinis LZP02 on rice roots (20). In this study, we hypothesized that citric acid promoted the colonization of B. altitudinis LZP02 on rice roots by inducing bacterial chemotaxis, thus improving the growth-promoting ability of B. altitudinis LZP02 on rice roots.

## RESULTS

### Effect of citric acid on the colonization of B. altitudinis LZP02 on rice roots.

**FIG 1:**
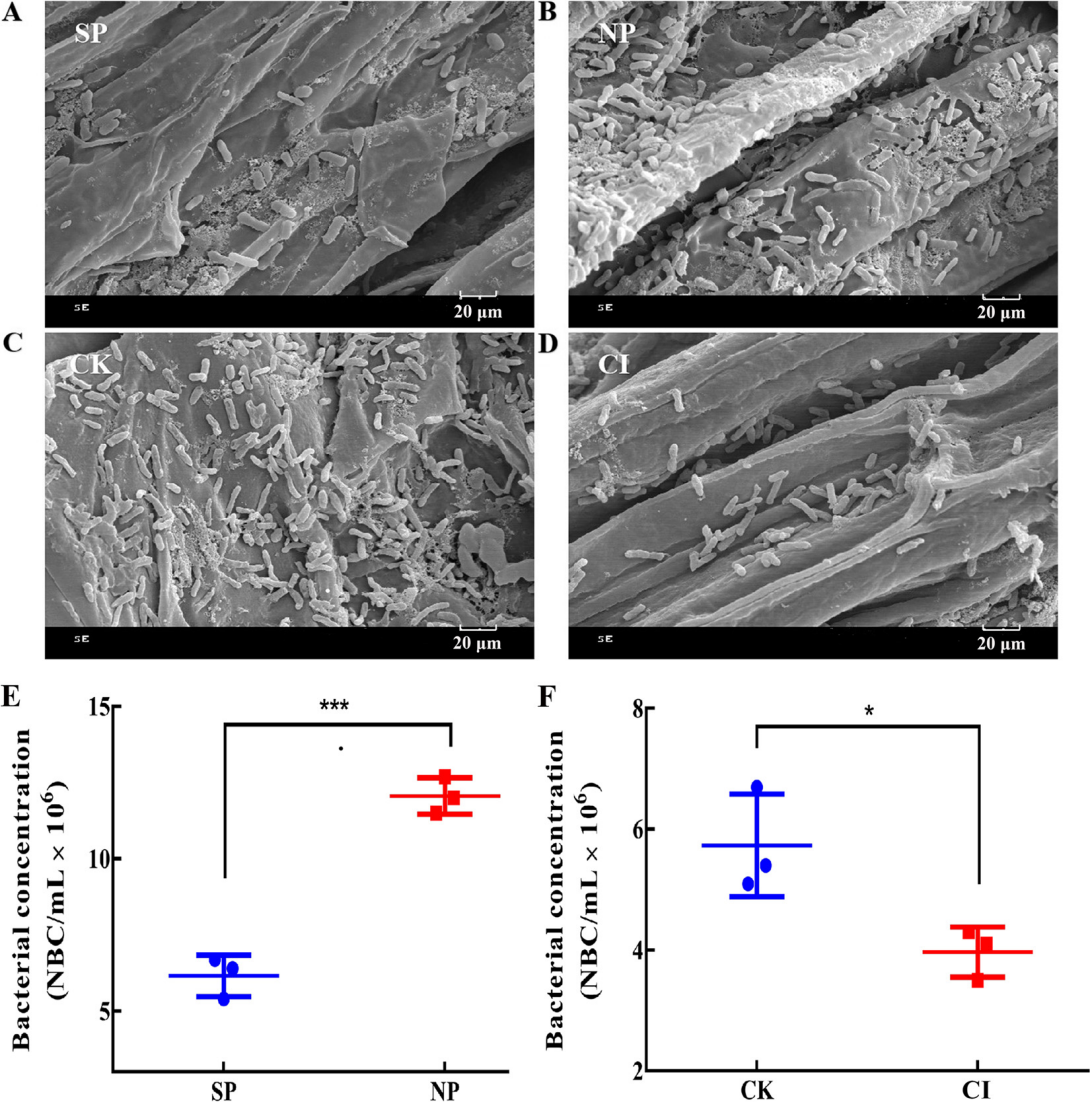
Scanning electron microscopy images of rice roots under 50,000× magnification and quantification of bacteria on the roots. (A) SP, the roots were inoculated with B. altitudinis LZP02 for 12 h after soaking in sterile water. (B) NP, the roots were inoculated with B. altitudinis LZP02 for 12 h after soaking in a 100 μM sterile citric acid aqueous solution. (C) CK, the roots were inoculated with sterile water for 24 h after soaking in a B. altitudinis LZP02 solution (OD = 0.5). (D) CI, the roots were inoculated with 100 μM sterile citric acid for 24 h after soaking in a B. altitudinis LZP02 solution (OD = 0.5). (E and F) The ordinates represent the bacterial concentrations, and the abscissas represent the treatments. *, *P < *0.05; and ***, *P < *0.001. According to *t* tests, *P *≤ 0.05 is considered significant.

The changes in the number of B. altitudinis LZP02 colonies on the rice roots are shown in Fig. 1. Scanning electron microscopy (SEM) images at 50,000× magnification showed that the NP treatment (roots inoculated with B. altitudinis LZP02 for 12 h after soaking in a 100 μM sterile citric acid aqueous solution) markedly increased the number of B. altitudinis LZP02 colonies compared with those under the SP treatment (roots inoculated with B. altitudinis LZP02 for 12 h after soaking in sterile water) (Fig. 1A and B). The number of B. altitudinis LZP02 colonies on rice roots was significantly reduced in the CI treatment (roots inoculated with 100 μM sterile citric acid for 24 h after soaking in a B. altitudinis LZP02 solution) compared with the CK treatment (roots inoculated with sterile water for 24 h after soaking in a B. altitudinis LZP02 solution) (Fig. 1C and D). The number of B. altitudinis LZP02 colonies in the NP treatment was 1.97-fold higher than that in the SP treatment (Fig. 1E). In addition, the number of B. altitudinis LZP02 colonies in the CI treatment was 1.46-fold lower than that in the CK treatment (Fig. 1F). These results confirmed that 100 μmol L^−1^ citric acid resulted in significant recruitment of B. altitudinis LZP02.

### Transcriptome analysis after citric acid treatment for 30 min.

**FIG 2:**
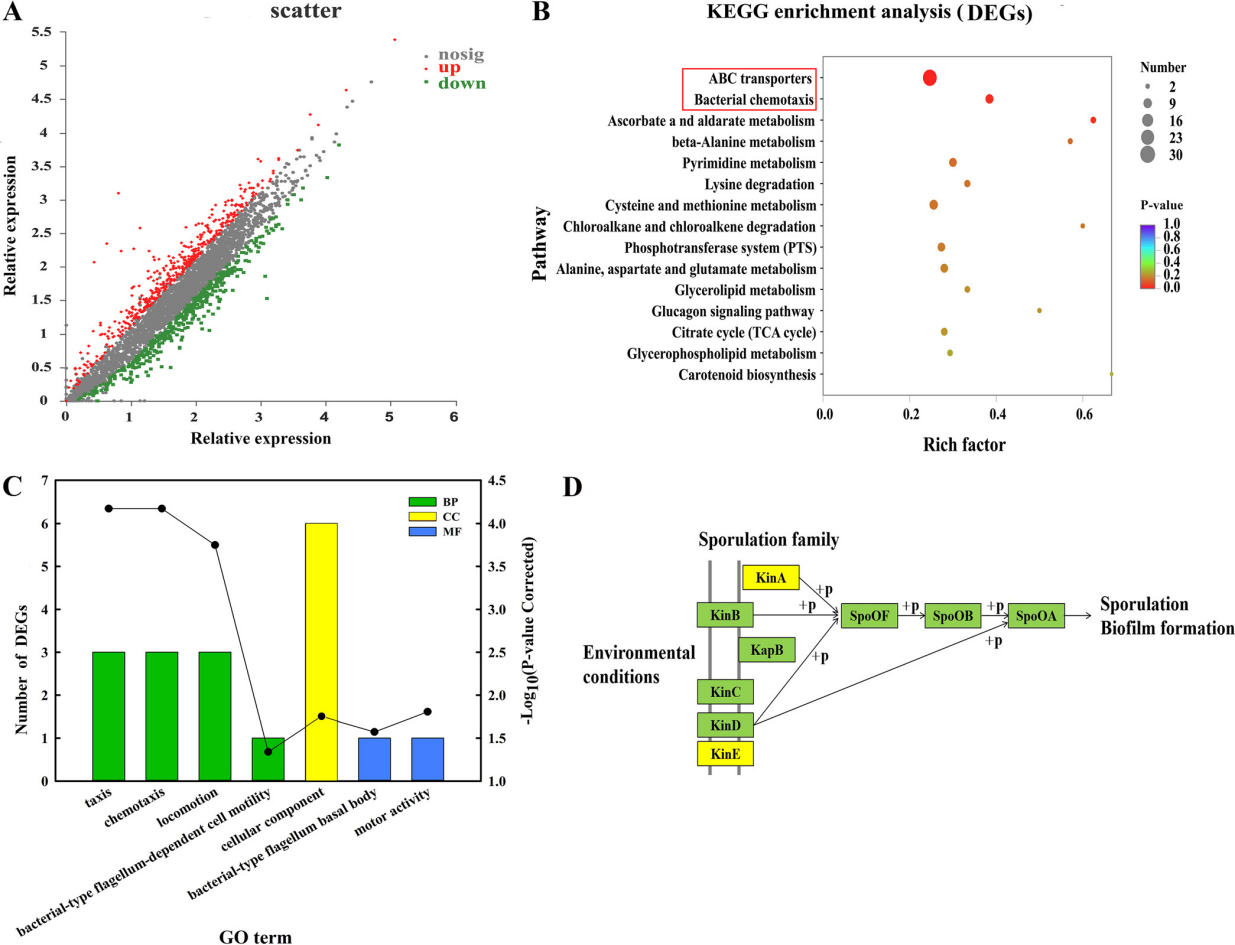
Transcriptome analysis of B. altitudinis LZP02 at 30 min after inoculation with citric acid. (A) Scatterplot of differentially expressed genes in the CK1 and CN1 treatments; the abscissas and ordinates represent the gene expression levels in the CK1 and CN1 treatments, respectively. Each dot represents a specific gene. (B) KEGG enrichment analysis of DEGs. (C) GO functional enrichment of DEGs. Significantly enriched (*P < *0.05) GO categories are shown. The abscissa represents the GO term, the left ordinate represents the number of DEGs (column), and the right ordinate represents the significance level of the enrichment (dot). The biological process (BP), cellular component (CC), and molecular function (MF) categories are represented by green, yellow and blue, respectively. (D) Biofilm formation process in B. altitudinis LZP02 in response to citric acid treatment. The yellow background color represents upregulated genes, while the green background color represents no differential expression.

The total number of differentially expressed genes (DEGs) detected in CN1 (sterile citric acid added to the B. altitudinis LZP02 solution to a final concentration of 100 μmol L^−1^) and CK1 (control treated with the same amount of sterile water) was 3,808. The number of upregulated and downregulated DEGs in CN1 was 428 and 405 (fold change ≥2 or ≤0.5; *P* ≤ 0.05), respectively, for a total of 833 genes differentially expressed between CK1 and CN1 (Fig. 2A). Kyoto Encyclopedia of Genes and Genomes (KEGG) pathway enrichment analysis was subsequently performed. As shown in a bubble diagram (Fig. 2B), ABC transporters and bacterial chemotaxis pathways were the most significantly enriched.

**FIG 3:**
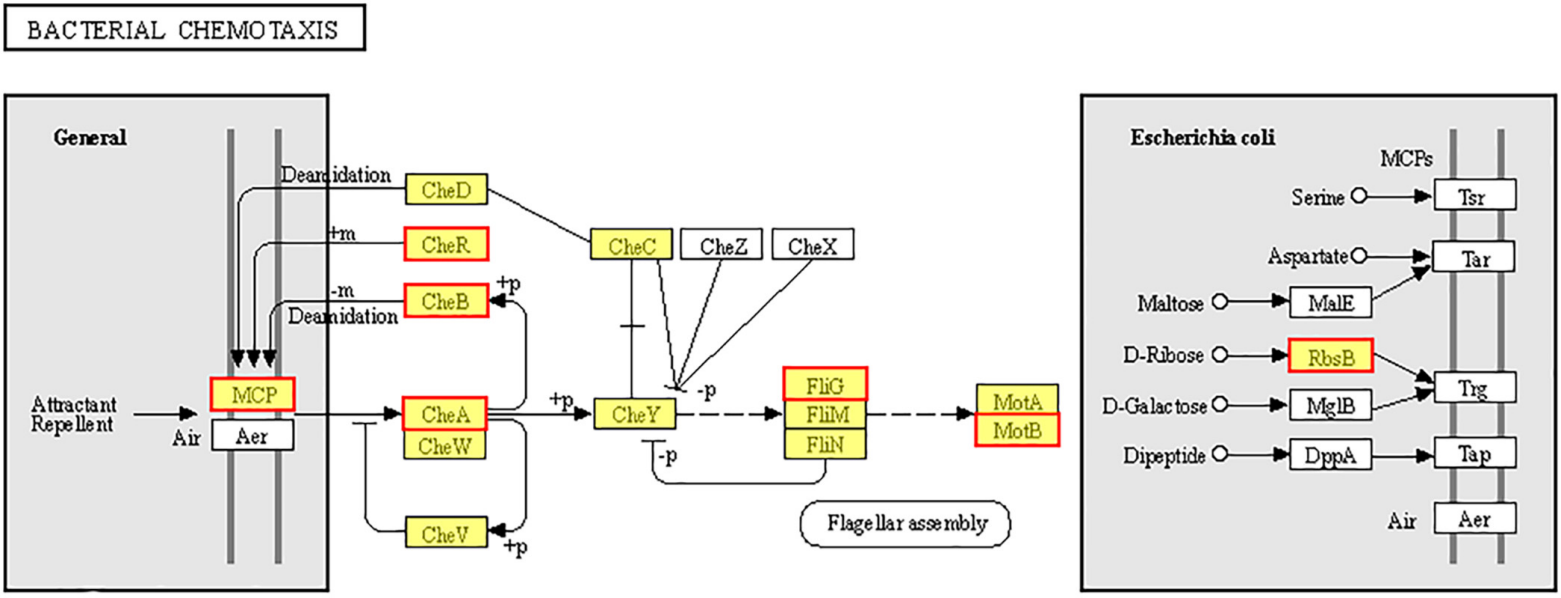
Bacterial chemotaxis pathway in B. altitudinis LZP02 in response to citric acid treatment. The figure was made in I-Sanger (https://cloud.majorbio.com/). The nodes associated with B. altitudinis LZP02 are shown in yellow. The red boxes represent upregulated genes. MCP, methyl-accepting chemotaxis proteins; CheR, PAS domain-containing protein; CheB, chemotaxis response regulator protein-glutamate methylesterase; CheA, chemotaxis proteins; FliG, flagellar motor switch protein; MotB, flagellar motor protein; RbsB, chemoreceptor for chemotaxis.

Gene Ontology (GO) enrichment analysis revealed 5 GO terms associated with bacterial chemotaxis and flagellar assembly (Fig. 2C). Specifically, there were 3, 3, 3, and 1 DEGs enriched taxis (GO:0044330), chemotaxis (GO:0006935), locomotion (GO: 0040011), and bacterial-type flagellum-dependent cell motility (GO:0071973), respectively; all of these genes were upregulated. The KEGG analysis showed that 10 DEGs associated with bacterial chemotaxis were upregulated (Table S1 in supplemental materials). Among their 7 protein products (Fig. 3), MCP is a methyl-accepting chemotaxis protein. It is worth noting that 3 *mcpA* and 1 *mcpC*, which are involved in MCP protein synthesis, were upregulated (Table S1). CheR is a Per-Arnt-Sim (PAS) domain-containing protein; CheB is a chemotaxis response regulator protein-glutamate methylesterase; CheA is a chemotaxis protein; FliG is a flagellar motor switch protein; MotB is a flagellar motor protein; and RbsB is a chemoreceptor for chemotaxis. Two DEGs were associated with biofilm formation, and both of these genes were upregulated (Fig. 2D). The results indicated that several genes associated with bacterial chemotaxis, flagellar assembly, and biofilm formation were affected by citric acid within a short amount of time. These results showed that citric acid increased the colonization ability of B. altitudinis LZP02 in the rice rhizosphere.

### Transcriptome analysis in response to citric acid treatment for 12 h.

**FIG 4:**
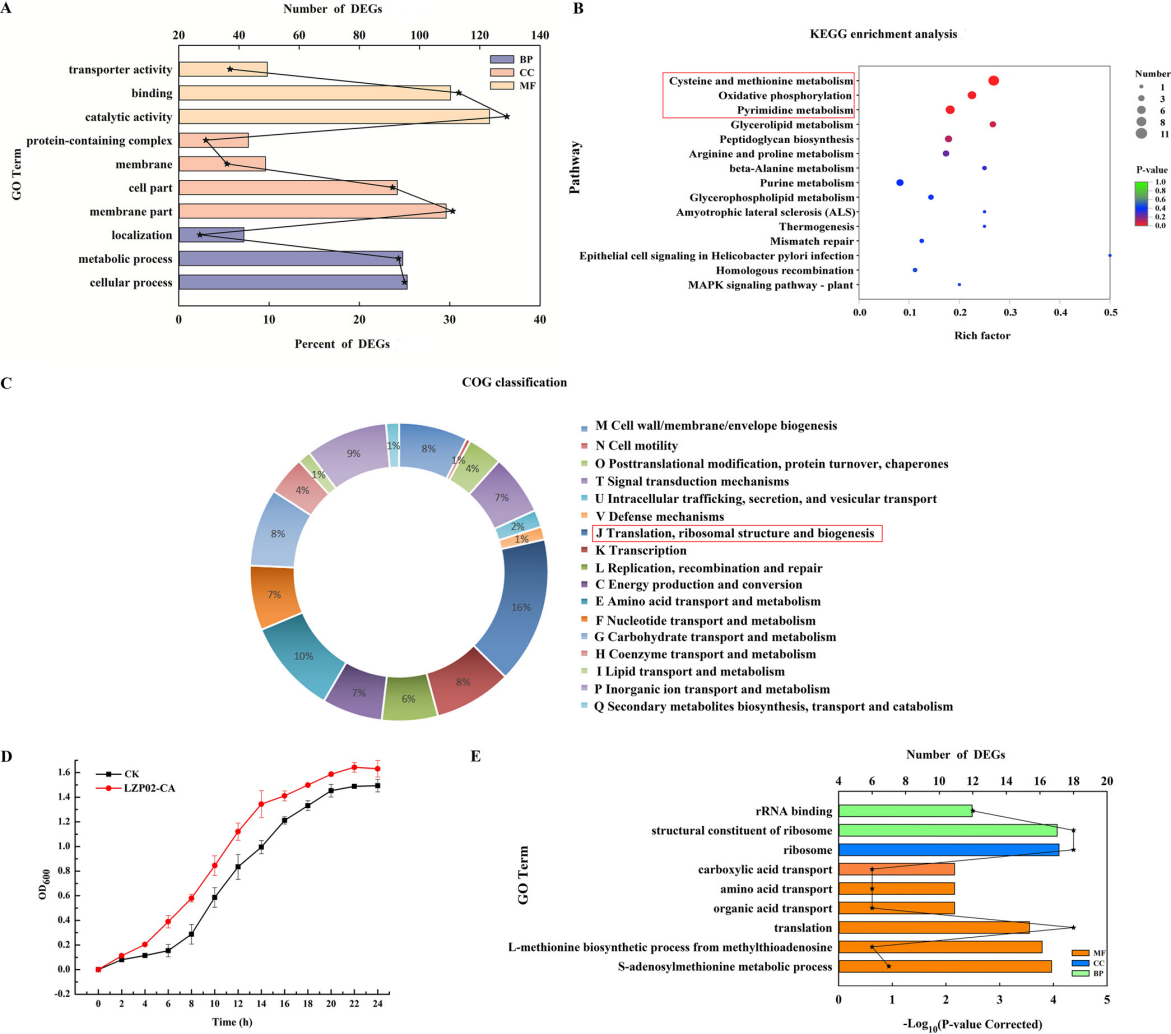
Transcriptome analysis of B. altitudinis LZP02 at 12 h after inoculation with citric acid and growth curve determination. (A) GO functional annotations of DEGs. The ordinate represents the GO term, the bottom abscissa represents the percent (column), and the upper abscissa represents the number of DEGs (dot). (B) KEGG enrichment analysis of DEGs. (C) COG functional annotations of DEGs. (D) Growth curves of B. altitudinis LZP02 and the bacteria treated with citric acid (LZP02-CA). (E) GO functional enrichment of DEGs. Significantly enriched (*P < *0.05) GO categories are shown in the figure. The ordinate represents the GO term, the bottom abscissa represents the significance level of enrichment (column), and the top abscissa represents the number of DEGs (dot).

GO functional annotation analysis was performed. The common DEGs were assigned to 30 GO terms (level 2) assigned to 3 different categories: 10 biological processes, 9 cellular components, and 11 molecular functions. As shown in Fig. 4A, the common DEGs were significantly enriched in the following top 10 terms: catalytic activity (GO:0003824), binding (GO:0005488), membrane part (GO:0044425), cellular process (GO:0009987), metabolic process (GO:0008152), cell part (GO:0044464), transporter activity (GO:0005215), membrane (GO:0016020), protein-containing complex (GO:0032991), and localization (GO:0051179). Moreover, a large percentage of DEGs were involved in catalytic activity (34.4%), which belongs to the molecular function category.

As shown in the results of the KEGG pathway enrichment analysis (Fig. 4B), the cysteine and methionine metabolism, oxidative phosphorylation, and pyrimidine metabolism pathways were significantly enriched (*P* < 0.05). In addition, 11, 7, and 8 DEGs were involved in three pathways; all these genes were significantly upregulated (Table S2).

The DEGs were classified via Clusters of Orthologous Groups (COG) functional annotation. The DEGs were involved in translation, ribosomal structure and biogenesis, amino acid transport, metabolism, inorganic ion transport, and metabolism and transcription (Fig. 4C; Table S3).

The results of the GO enrichment analysis are shown in Fig. 4E. In the biological process category, 12 and 18 DEGs were associated with rRNA binding (GO:0019843) and structural constituents of the ribosome (GO:0003735), respectively. In the cellular components category, 18 DEGs were associated with the ribosome (GO: 00056840). In the molecular function category, 6, 6, 6, 18, 6, and 7 DEGs were associated with carboxylic acid transport (GO:0046942), amino acid transport (GO:0006865), organic acid transport (GO:0015849), translation (GO:0006412), the l-methionine biosynthetic process from methylthioadenosine (GO:0019509), and the *S*-adenosylmethionine metabolic process (GO:0046500), respectively. All these terms were significantly enriched. Information about these genes is included in Table S4.

The results indicated that citric acid mainly affects the expression of genes involved in organic acid transport, transcription, translation, l-methionine biosynthesis, *S*-adenosylmethionine metabolism, and oxidative phosphorylation in the bacteria after 12 h.

### Effects of citric acid on the growth of B. altitudinis LZP02.

To assess the influence of citric acid on B. altitudinis LZP02, the growth of B. altitudinis LZP02 in the presence of 100 μM citric acid was analyzed. As shown in Fig. 4D, the growth of B. altitudinis LZP02 in the LZP02-CA treatment was obviously different from that in the control. The bacterial trains entered the logarithmic phase earlier, and the optical density (OD) value was greater.

### Transcriptome differences in response to citric acid treatment at 30 min and 12 h.

**FIG 5:**
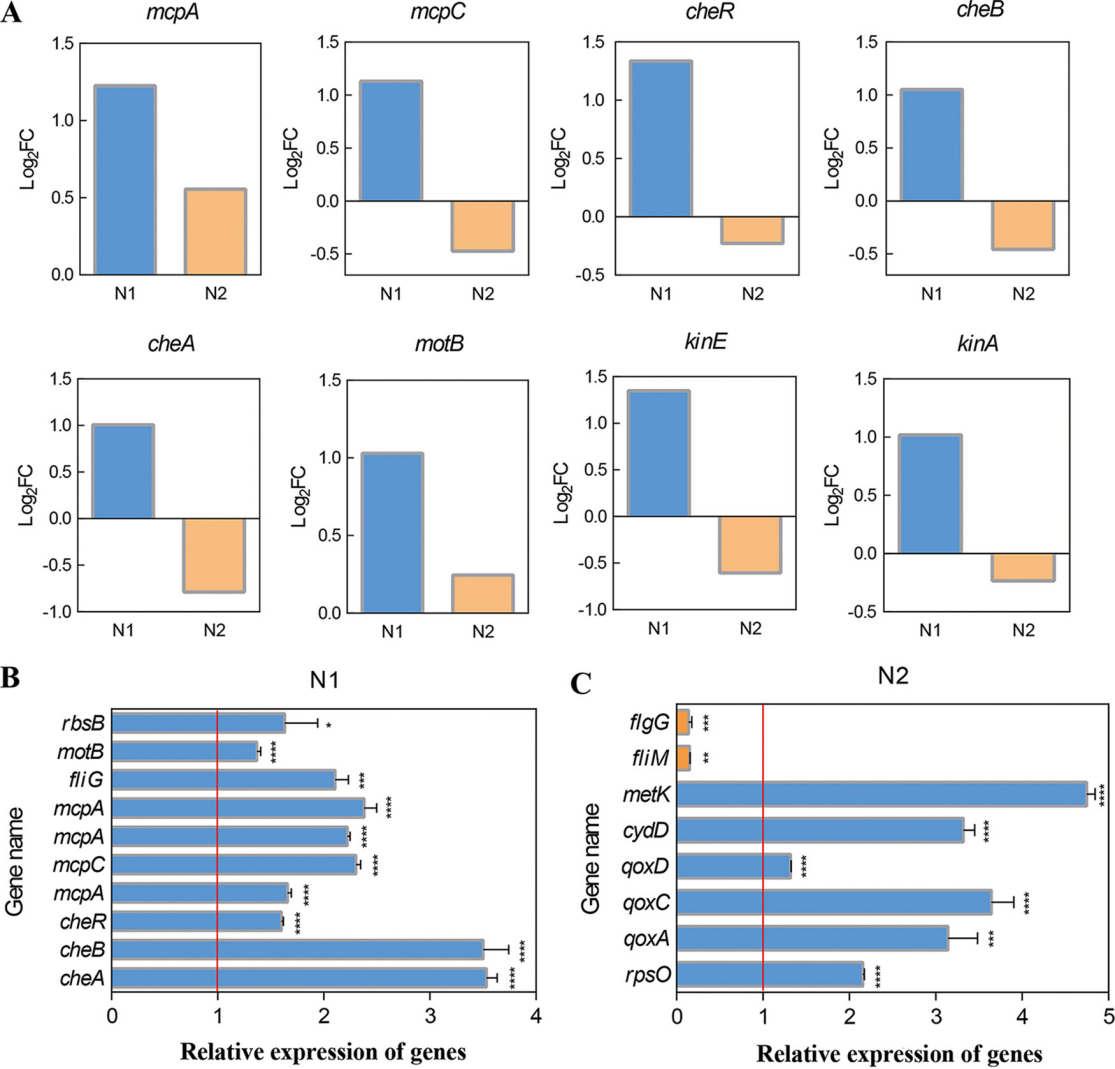
Transcriptome differences in response to citric acid for 30 min (N1) and 12 h (N2) and validation of RNA-seq data via qRT-PCR. (A) Comparison of log_2_(FC) values of genes associated with bacterial chemotaxis and biofilm formation in N1 and N2. (B, C) Expression of differentially expressed genes according to qRT-PCR. Data <1 indicate that a gene is downregulated, and data >1 indicate gene is upregulated. *, *P < *0.05; **, *P < *0.01; ***, *P < *0.001; and ****, *P < *0.0001. According to *t* tests, *P ≤ *0.05 is considered significant.

Many of the DEGs in the bacterial strain were upregulated in response to citric acid treatment at different treatment times. The upregulated DEGs were involved in different pathways, but the expression of genes involved in the same pathway was different. As shown in Fig. 5A, 6 genes (*mcpA*, *mcpC*, *cheR*, *cheB*, *cheA*, and *motB*) were involved in the bacterial chemotaxis pathway, and 2 genes (*kinE* and *kinA*) were associated with biofilm formation. According to the transcriptome results of N1 (30-min citric acid treatment), these genes were significantly upregulated (log_2_[FC] > 1). According to the transcriptome results of N2 (12-h citric acid treatment), 2 genes associated with bacterial chemotaxis (*mcpA* and *motB*) were insignificantly upregulated. Four genes associated with bacterial chemotaxis (*mcpC*, *cheR*, *cheB*, and *cheA*) were statistically insignificantly downregulated. Two genes associated with biofilm formation (*kinE* and *kinA*) were statistically insignificantly downregulated.

### Reverse transcription-quantitative PCR analysis.

To confirm the RNA-seq data, the expression of several genes, including 10 DEGs in N1 and 8 DEGs in N2, was determined. The expression levels of *flgG* and *fliM* in N2 were significantly decreased in citric acid-treated bacteria, and the expression levels of the other 16 DEGs were significantly increased. These results are consistent with the results of the transcriptomic analysis (Fig. 5B, C; Tables S1, S2, and S4).

### Induction of citric acid production in rice roots.

**FIG 6:**
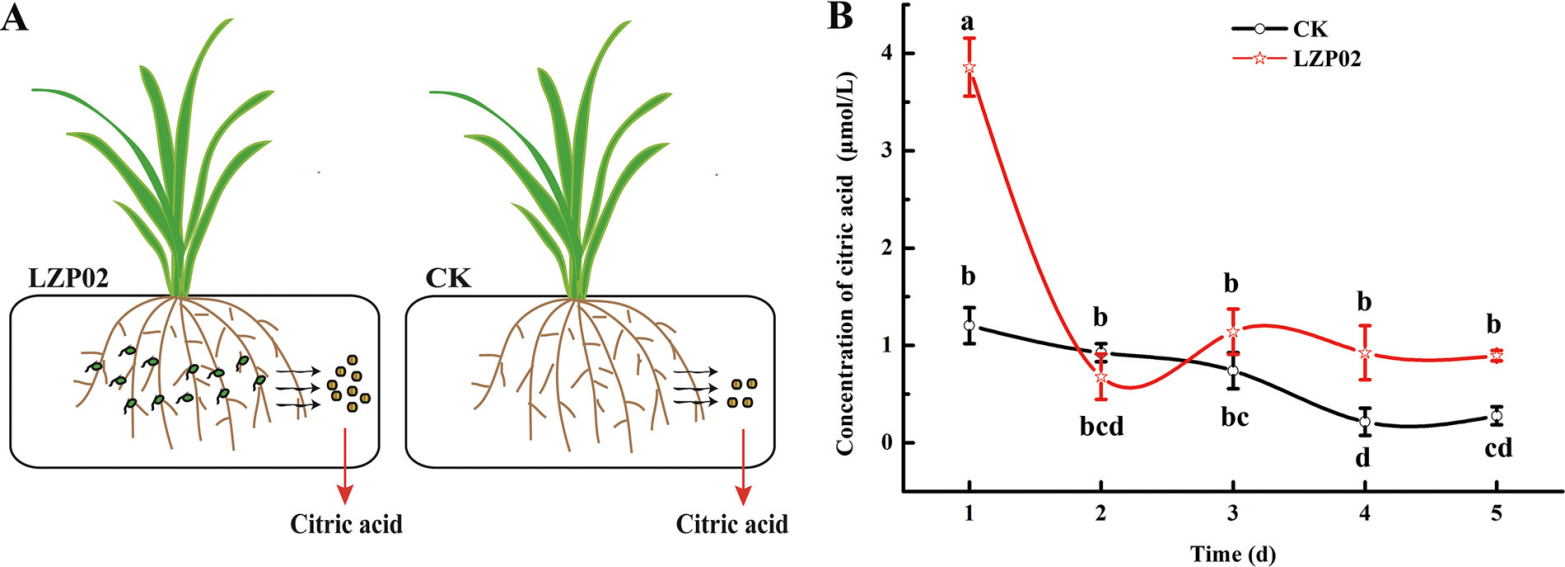
Quantification of citric acid concentrations in root exudates. (A) Schematic diagram of sample collection. (B) Quantification of citric acid concentrations was performed using a CA kit. The error bars are the standard errors of 3 experimental replicates.

The concentration of citric acid in the plant rhizosphere provides chemoattractant functions and induces biofilm formation. The concentration of citric acid in the rice root exudates was measured after incubation for 1 to 5 days. The results showed that root exudates from the uninoculated rice (CK treatment) produced 1.20 μmol L^−1^ citric acid in 1 day; however, LZP02 treatment resulted in 3.85 μmol L^−1^ citric acid in 1 day, which was 3 times higher than that of the control. At 3 to 5 days, the concentration of citric acid was significantly increased in the LZP02 treatment compared with the CK (Fig. 6B). These results indicated that the secretion of citric acid was induced by B. altitudinis LZP02. Taken together, these findings suggest that citric acid might be an important signal by root exudates.

### Plant growth-promoting trait analysis.

**FIG 7:**
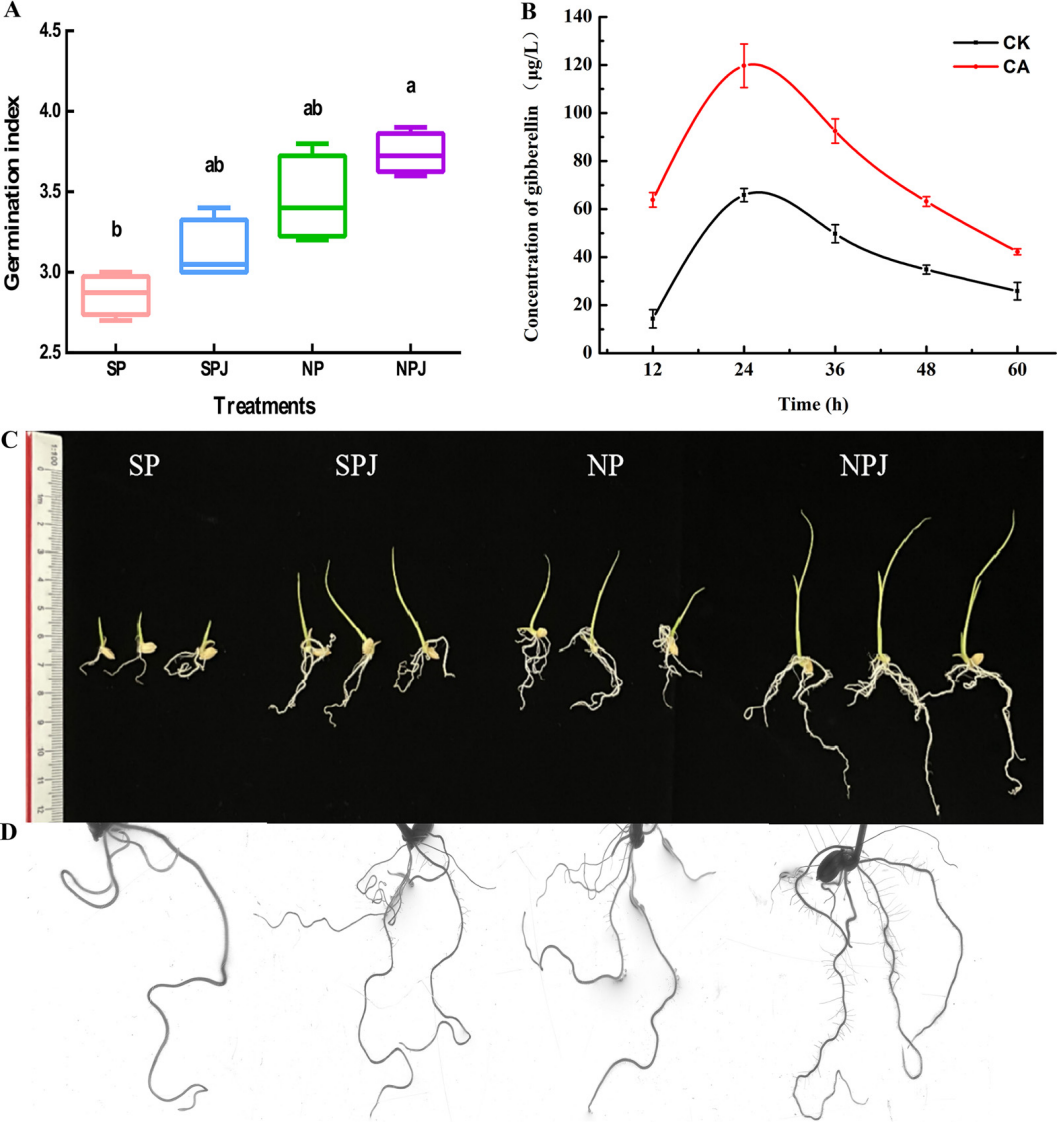
Germination index and growth parameters of rice were observed and determined, and the effects of citric acid on the GA secretion of B. altitudinis LZP02 was measured. SP: control group; SPJ, B. altitudinis LZP02; NP, citric acid; NPJ, combined treatment of citric acid and B. altitudinis LZP02. (A) Determination of the germination index. (B) Quantification of GA secretion of B. altitudinis LZP02. (C) Rice growth. (D) Scanned images of rice roots. The error bars was the standard errors of three experimental replicates. The columns denoted by different letters are significantly different at *P ≤ *0.05.

The germination index of rice was determined. As shown in Fig. 7A, the germination index of rice increased gradually across the 4 treatments. In addition, the germination index of rice was significantly increased in the combined treatment of citric acid and B. altitudinis LZP02 (NPJ) compared with the SP treatment. The gibberellin (GA) secretion of B. altitudinis LZP02 treated with citric acid (CA treatment) was significantly increased compared with that under the CK treatment (Fig. 7B). The effect of citric acid and B. altitudinis LZP02 on the growth of rice was subsequently explored. The growth-promoting effects of these treatments were different from those of the SP treatment. Compared with the SP treatment, the NPJ treatment resulted in the highest growth. Scans of the rice roots revealed that the 8 parameters of the roots of rice in the NPJ treatment increased significantly. The effect of NP treatment was also relatively greater because citric acid can increase the germination index of rice and shorten the germination time of seeds (Fig. 7C). The results showed that the combined treatment of citric acid and B. altitudinis LZP02 promoted rice growth.

**FIG 8:**
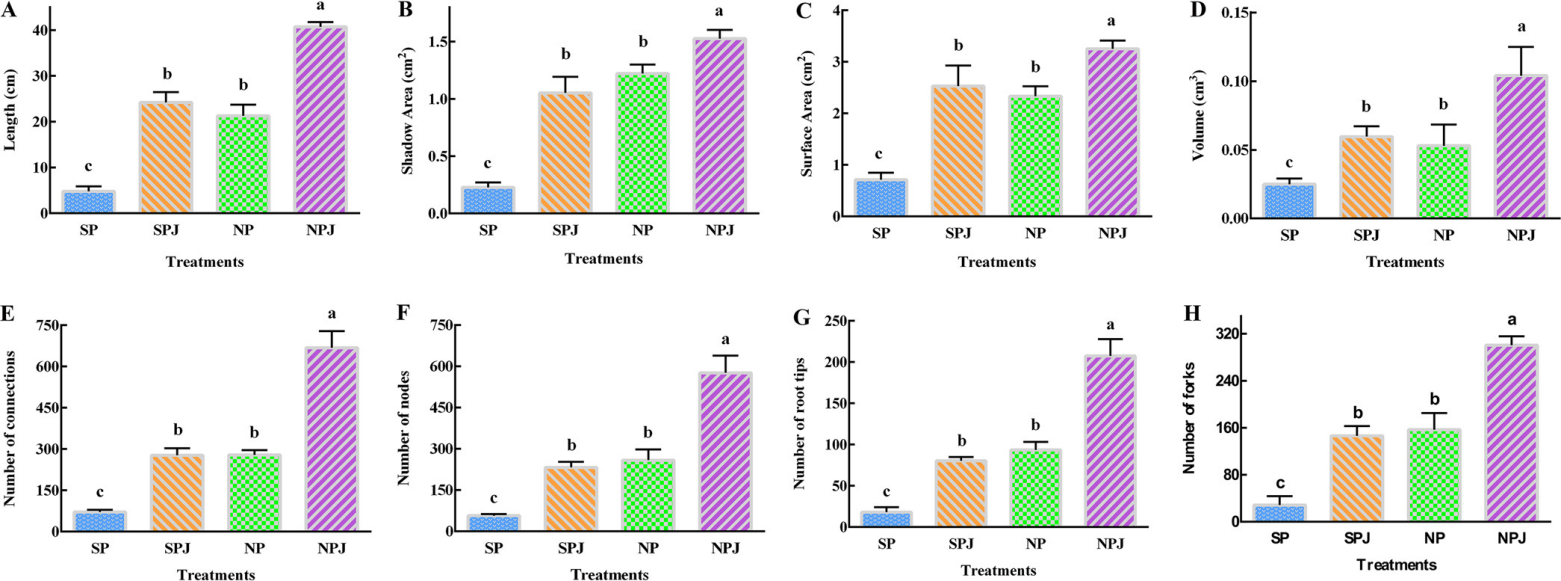
Analysis of rice biological characteristics. The detailed 8 root parameters is described. SP, control group; SPJ, B. altitudinis LZP02; NP, citric acid; NPJ, combined treatment of citric acid and B. altitudinis LZP02. The columns denoted by different letters are significantly different at *P ≤ *0.05.

Eight growth traits were measured under different conditions. The growth traits included root length (Fig. 8A), shadow area (Fig. 8B), surface area (Fig. 8C), root volume (Fig. 8D), number of connections (Fig. 8E), number of nodes (Fig. 8F), number of root tips (Fig. 8G), and number of forks (Fig. 8H). Compared with those under the SP treatment, the root length, shadow area, surface area, root volume, number of connections, number of nodes, number of root tips, and number of forks under the SPJ treatment (10 mL of B. altitudinis LZP02 plus sterile water added to the water-soaked seeds) increased by 5.05-fold, 4.63-fold, 3.54-fold, 2.39-fold, 3.90-fold, 4.08-fold, 4.46-fold, and 5.16-fold, respectively; those under the NP treatment increased by 4.44-fold, 5.38-fold, 3.27-fold, 2.12-fold, 3.92-fold, 4.54-fold, 5.18-fold, and 5.29-fold, respectively; and those under the NPJ treatment increased by 8.50-fold, 6.72-fold, 4.56-fold, 4.17-fold, 9.40-fold, 10.11-fold, 11.51-fold, and 10.61-fold, respectively. The experiment showed that rice root growth was enhanced under the SPJ, NP, and NPJ treatments. Notably, the effect of rice growth promotion was the most significant under the combined treatment of citric acid and B. altitudinis LZP02.

## DISCUSSION

Organic acids in plant root exudates have a significant effect on root colonization of PGPR and increase the occurrence of plant-microbe interactions (21, 22). The present study highlights the importance of citric acid in plant growth-promoting rhizobacterium. B. altitudinis LZP02 is a beneficial plant growth-promoting rhizobacterium with a strong ability of rice rhizosphere colonization and promote rice growth (23). Our previous study showed that the citric acid within rice root exudates induced the chemotaxis of B. altitudinis LZP02, and 100 μM was determined to be the optimal concentration for bacterial chemotaxis (20). Here, we concentrated on the interaction between citric acid and B. altitudinis LZP02 at the molecular level and on rice growth-promoting trait analysis. In this study, SEM clearly indicated that the introduction of citric acid could increase the number of B. altitudinis LZP02 colonies in the rice roots. These results were confirmed by reverse verification, such that when citric acid was added to the culture media, the bacteria on the roots collapsed. The quantification results were consistent with the observations from the SEM images (Fig. 1). These results confirmed that 100 μmol L^−1^ citric acid can lead to significant recruitment of B. altitudinis LZP02. In agricultural production, rice roots secrete citric acid, which induces chemotaxis of bacterial strains in the soil. The results demonstrated that citric acid in rice root exudates enhanced the colonization of the bacterial strain LZP02.

Flagellum-mediated motility plays an important role in various physiological activities, such as bacterial chemotaxis, biofilm formation, and host colonization (24–27). We further revealed that citric acid considerably increased the expression of genes related to chemotaxis and flagellar biosynthesis in B. altitudinis LZP02, thus enhancing its motility (20) and colonization in the rice root (Fig. 2). In this study, KEGG enrichment analysis of the transcriptome discovered that 12 DEGs associated with bacterial chemotaxis and biofilm formation were significantly upregulated in B. altitudinis LZP02 after 30 min of citric acid treatment (Table S1). These genes encode 8 proteins (e.g., MCP, a methyl-accepting chemotaxis protein; FliG, a flagellar motor switch protein; and MotB, a flagellar motor protein) (Fig. 3 and Table S1). The phenotypic results were consistent with the results of the transcriptome analysis. Bacterial chemotaxis depends on MCP sensing and binding to chemoeffectors, followed by transmitting the perceived signal to downstream proteins that play a role in the chemotaxis signaling system (28). It is well known that this process is rapid (29). We found that the expression of these genes associated with bacterial chemotaxis and biofilm formation differed in B. altitudinis LZP02 after 12 h of citric acid treatment (Fig. 5). These findings supported the results of the transcriptome analysis after 30 min of citric acid treatment. Similarly, another study found that genes involved in quorum sensing, chemotaxis, and biofilm formation in Paenibacillus polymyxa SC2 were significantly upregulated after pepper root exudate treatment for 30 min (30).

A previous study showed that d-galactose from cucumber roots could induce biofilm formation of B. velezensis SQR9 in an *mcpA*-dependent manner (31). Moreover, flagellum-related genes were significantly upregulated in Pseudomonas koreensis GS after treatment with Streptomyces pactum Act12 cell-free filtrate, while rhizosphere colonization and competition were enhanced (32). A previous study found that a *motA*-deficient strain showed lower colonization ability in wheat roots, while the biocontrol effects that worked against it were reduced (33). Chemotaxis is the first step during rhizosphere colonization. These results confirmed that an increase in the expression of flagellum-mediated motility genes after citric acid treatment may promote root colonization by B. altitudinis LZP02 in the rice rhizosphere.

Competition is more prevalent in free-living microbes in habitats where resources are relatively scarce, thus improving the physiological activity of bacteria and supporting the impact of plant growth promotion. Here, the bacteria entered the logarithmic phase earlier after citric acid treatment, and the OD value was larger (Fig. 4D). Soil microorganisms confirmed rapid and complete metabolism with citric acid as a source of carbon and energy. Biostimulation of citric acid was confirmed, which showed an increase in dehydrogenase and phosphatase activities (34). *S*-adenosylmethionine (SAM) is an important physiologically active substance. As a methyl donor, aminopropyl donor and precursor of sulfhydryl compounds, SAM is involved in a range of biochemical reactions in all living organisms (35). Examples include the synthesis of nucleic acids, proteins, phospholipids, and vitamins and the mutual transformation of cysteine, glutathione, polyamines, and taurine (36–38). Our GO enrichment analysis of the transcriptome data showed that 7 DEGs associated with *S*-adenosylmethionine biosynthesis and metabolism were significantly upregulated in B. altitudinis LZP02 after 12 h of citric acid treatment (Fig. 4E and Table S4). According to a previous study, SAM is known to participate in a number of essential metabolic pathways in plants (39). Whether such interaction mechanisms occur in the rhizosphere remains unknown. In vivo, SAM is formed by the transfer of an adenosine group of ATP to l-methionine, catalyzed by *S*-adenosylmethionine synthase (*metk*), which depletes ATP in the cells. Therefore, the level of intracellular ATP is one of the factors limiting the biosynthesis of SAM (36, 40). It is worth noting that 7 DEGs involved in the oxidative phosphorylation pathway were significantly upregulated in B. altitudinis LZP02. The results showed that the intracellular ATP level of B. altitudinis LZP02 significantly increased after treatment with citric acid for 12 h (Fig. 4B and Table S2). Therefore, citric acid might improve the physiological activity of bacteria by increasing the expression of genes involved in SAM biosynthesis and metabolism.

Moreover, B. altitudinis LZP02 caused an increase in the secretion of citric acid from the rice roots (Fig. 6B). These results indicate that citric acid might be a momentous signal in the interaction between plants and B. altitudinis LZP02, which is worthy of further study (31). Similarly, the d-galactose content was shown to be 3-fold higher in the root exudates of B. velezensis SQR9-colonized cucumber than in those of uninoculated cucumber (31), and B. subtilis RR4 isolated from the rice rhizosphere induces malic acid biosynthesis in rice roots (11). Our experiments showed that citric acid secretion in rice decreased after 2 days of B. altitudinis LZP02 treatment. We speculate that higher concentrations of citric acid were utilized by B. altitudinis LZP02 (Fig. 6).

Finally, the combined use of citric acid and B. altitudinis LZP02 treatment had a synergistic effect on plant growth promotion, including on both seed germination and growth traits, as observed during our experiments. The results showed that citric acid alone increased germination and plant growth. Additionally, the application of CA has been reported to enhance tomato seed germination and root length (41). However, the mechanism underlying the effect of low-molecular-weight organic acids on plant seeds is worthy of further study. Seed germination is the first step to ensuring grain yield and quality (42–44). A sufficient germination index and postgermination growth promote subsequent cultivation (45, 46). Moreover, bioactive GA promotes seed germination in a number of plant species (47, 48). Our results showed that citric acid promotes GA secretion by B. altitudinis LZP02. When rice seeds were soaked in citric acid, the addition of B. altitudinis LZP02 increased the germination index and enhanced rice growth traits (Fig. 7 and 8).

### Conclusion.

**FIG 9:**
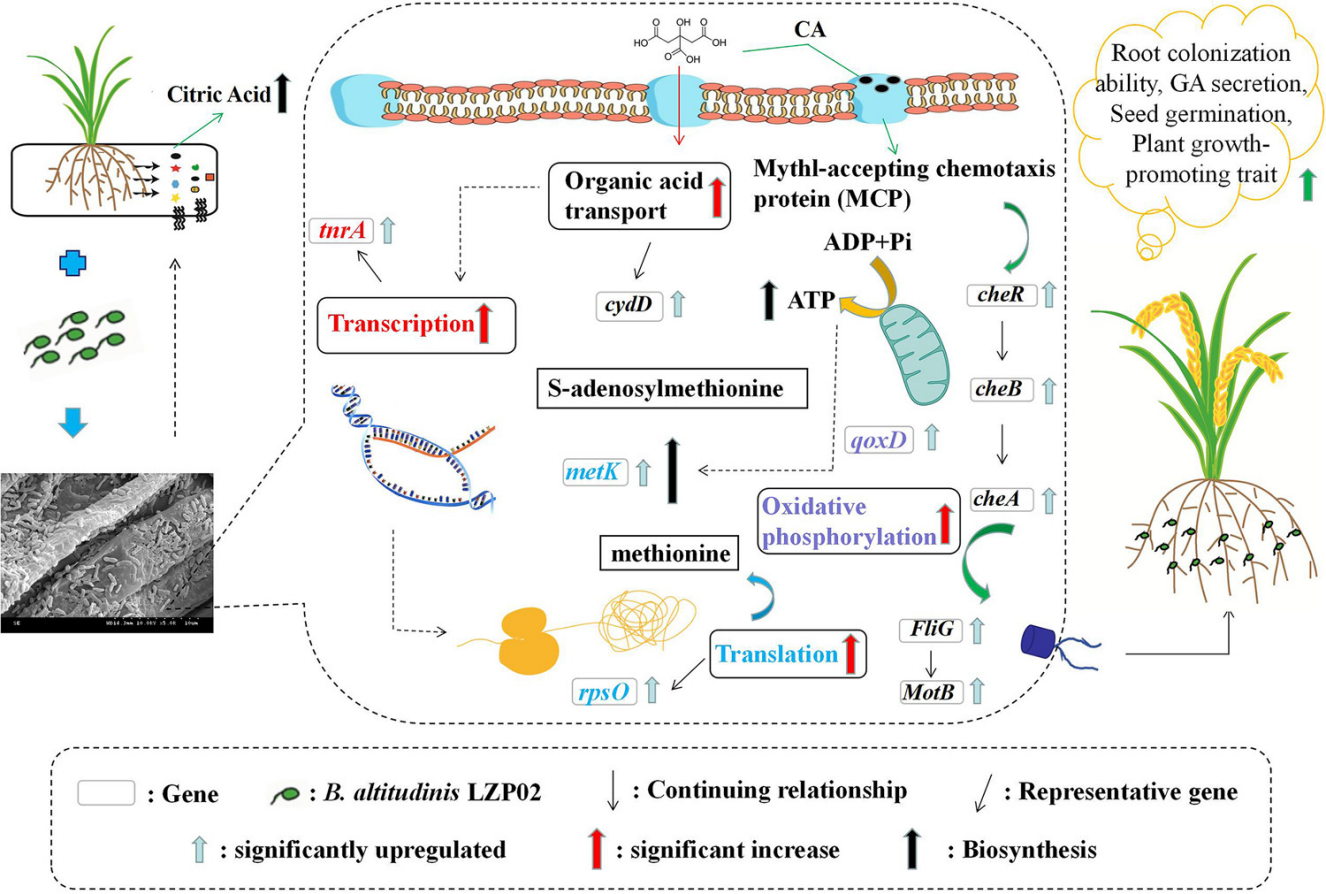
Schematic diagram of the mechanism underlying the interaction between citric acid and B. altitudinis LZP02. The dashed arrows indicate predicted processes.

A schematic diagram of the interaction between citric acid and B. altitudinis LZP02 is shown in Fig. 9. The citric acid in rice root exudates enhanced the colonization of B. altitudinis LZP02 by increasing the expression of genes involved in the bacterial chemotaxis and biofilm formation pathways. The citric acid promotes GA secretion by B. altitudinis LZP02. The combination of both citric acid and B. altitudinis LZP02 promoted seed germination and rice growth. Moreover, B. altitudinis LZP02 increased the secretion of citric acid from the rice roots. The results confirmed that the interaction between citric acid and B. altitudinis LZP02 could be exploited for the development of sustainable agriculture.

## MATERIALS AND METHODS

### Bacterial strain, media, plant material, and growth conditions.

B. altitudinis LZP02 is a rice rhizobacterium (GenBank: CP075052). B. altitudinis LZP02 was grown at 30°C in Luria-Bertani (LB) media (peptone, 10 g L^−1^; yeast extract, 5 g L^−1^; and NaCl, 8 g L^−1^) for 10 h, collected via centrifugation at 9,569 × *g*, and stored in 15% glycerol at −80°C. Rice seeds (Oryza sativa “Longgeng 46”) were used in this study. Murashige-Skoog (MS) liquid media was purchased from Sigma ALDRICH (Shanghai) TRADING Co., LTD.

### SEM.

Rice seedlings roots of a similar size were rinsed with sterile water. The roots were soaked in 100 μmol L^−1^ sterile citric acid aqueous solution for 9 h (NP). The roots of the control group were soaked with an equal volume of sterile water (SP). All the seedlings were placed in MS liquid media (50 mL) that included B. altitudinis LZP02 (4 × 10^7^ CFU/mL) for 12 h. In another contrasting experiment, the roots of seedlings were soaked in B. altitudinis LZP02 solution (OD_600_ = 0.5) for 1 h. The treated seedlings were subsequently transferred to MS liquid media. Sterile citric acid solution was added to the MS liquid media to a final concentration of 100 μmol L^−1^ (CI), and the control group was treated with the same amount of sterile water (CK). All the seedlings were transplanted into the media for 24 h. All of the operations were performed under temperature (22 ± 2°C). SEM was performed as previously described (49). The length of the root segments was 0.4 to 1 cm, and the root segments were fixed in 2.5% glutaraldehyde for 12 h. Then, a 40%, 50%, 60%, 70%, 80%, 90%, and 100% ethanol series was used to dehydrate the root segments, which were then allowed to dry under natural conditions. Two- to three-millimeter-thick samples were prepared. The different parts of roots were observed, and images were obtained (S-3400; Hitachi, China). Fibrous roots (0.01 g) of the same size were used in the above-described experiment. The samples were then placed in 1 mL of sterile water, vortexed for 30 min, and counted with a hemocytometer.

### Growth curve assays.

Growth curves of B. altitudinis LZP02 (CK) were constructed, and the control strains and strains treated with 100 μmol L^−1^ citric acid (LZP02-CA) in LB media were measured under the same conditions (120 rpm, 30°C) with shaking. Absorbance measurements at 600 nm were performed every 2 h. Three replicates were included for each treatment.

### Transcriptome analysis of B. altitudinis LZP02.

**(i) Sample preparation.** For this experiment, B. altitudinis LZP02 was treated with sterile citric acid for 30 min. The steps for sample preparation included B. altitudinis LZP02 being grown at 30°C in media (peptone, 10 g L^−1^; NaCl, 8 g L^−1^) to an OD = 0.7. In the next step, sterile citric acid was added to the B. altitudinis LZP02 solution to a final concentration of 100 μmol L^−1^ (CN1), while the control was treated with the same amount of sterile water (CK1). The treated bacteria were centrifuged after the culture oscillated for 30 min.

In addition, B. altitudinis LZP02 was treated with sterile citric acid for 12 h. The steps for sample preparation included the addition of sterile citric acid to the media (peptone, 10 g L^−1^; NaCl, 8 g L^−1^) to a final concentration of 100 μmol L^−1^ (CN2), while the control received an equal volume of sterile water (CK2). Each treatment was inoculated with 1 mL of broth culture of B. altitudinis LZP02 for 8 h. The culture was incubated at 30°C and shaken at 120 rpm for 12 h. The bacterial cells in each group were harvested by centrifugation (8,000 rpm, 4°C), and samples were sent directly to Majorbio Biopharm Technology Co., Ltd. (Shanghai, China) for transcriptome sequencing.

**(ii) RNA extraction, library construction, and sequencing.** Total RNA was extracted using TRIzol reagent (Invitrogen) and quantified using an ND2000 (NanoDrop Technologies). Only high-quality RNA (OD_260/280_ = 1.8~2.0; OD_260/230_ ≥2.0; relative intensity noise ≥6.5; 28S:18S ≥1.0; ≥100 ng/μL; and ≥2 μg) was used to construct the sequencing library.

The RNA-seq library was prepared according to instructions of the TruSeq RNA sample preparation kit from Illumina (San Diego, CA), with 2 μg of total RNA used. Double-stranded cDNA was synthesized and subjected to end repair, phosphorylation, and polyadenylation. The libraries were screened for cDNA target fragments of 200 bp followed by PCR amplification of 15 PCR cycles. After quantification via TBS380, the paired-end RNA-seq library was sequenced by an Illumina HiSeq×10 instrument (2 × 150-bp read length).

**(iii) Bioinformatics analysis.** High-quality reads in each sample were aligned to the reference genome of B. altitudinis LZP02 (this study; NCBI accession number CP075052) using Bowtie2 (50, 51). The expected number of fragments per kilobase of transcript per million base pairs sequenced of each gene was calculated (52).

The edgeR (http://www.bioconductor.org/), DESeq2 (http://bioconductor.org/), and DESeq packages (http://www.r-project.org/) were used to identify DEGs across samples or groups (53, 54). The genes with a fold change ≥2 and a *P* < 0.05 in each comparison were regarded as significant DEGs (55). GO enrichment analysis of DEGs was performed using GOATOOLS (https://github.com/tanghaibao/GOatools) (56). The statistical enrichment of DEGs in KEGG pathways was determined using KOBAS 2.0 software (https://doi.org/10.18170/DVN/ZKCO43) (57).

### Validation of transcriptome data by reverse transcription-quantitative PCR.

To verify the RNA-seq results, 18 DEGs from the RNA-seq analysis were selected, and reverse transcription-quantitative PCR (qRT-PCR) was performed to confirm their expression changes. Moreover, qRT-PCR was performed on the RNA samples of RNA-seq. Single-stranded cDNA was generated from the total RNA using HiScript (Vazyme) followed by quantitative real-time PCR (ABI7300; Applied Biosystems, USA). The primers and internal control used for qRT-PCR are listed in Table S5 and Table S6. Each qRT-PCR mixture (20 μL) included 10 μL of 2× ChamQ SYBR Color qPCR Master Mix, 0.8 μL of forward primer (5 μM), 0.8 μL of reverse primer (5 μM), 0.4 μL of 50× ROX Reference Dye 1, 2 μL of template (cDNA), and 6 μL of ddH_2_O. PCR was performed as follows: 5 min at 95°C followed by 40 cycles of 5 s at 95°C and 30 s at 55°C. This study was performed by using the 2^−^^ΔΔ^^Ct^ method (58). Each measurement was repeated three times.

### Quantitation of citric acid concentration.

Sterile rice seeds were cultivated until the seedling stage was reached. Rice seedlings of similar size were transplanted into MS liquid medium (50 mL). In the treatment group, the media for B. altitudinis LZP02 were washed away and added to MS liquid media to a final concentration of 4 × 10^7^ CFU/mL, while the control group received an equal volume of sterile water. Three replicates were included for each treatment. The citric acid concentration in rice roots was measured using test kits (Nanjing Jiancheng Biological Engineering Institute, Nanjing, China). In this experiment, rice seedlings were grown in a growth chamber; the roots were inoculated with B. altitudinis LZP02, and the root exudates were collected for quantitative analysis (Fig. 6A).

### Analysis of plant growth-promoting traits.

The rice seeds were disinfected with H_2_O_2_ and rinsed six times with sterile water.

The sterilized seeds were soaked in a 100 μmol L^−1^ citric acid solution and sterile water for 2 h each. Then, the seeds were transferred to the growth chamber. Ten milliliters of B. altitudinis LZP02 (4 × 10^7^ CFU/mL) (NPJ) plus sterile water (NP) was added to the seeds soaked in citric acid. In addition, 10 mL of B. altitudinis LZP02 (4 × 10^7^ CFU/mL) (SPJ) plus sterile water (SP) was added to the water-soaked seeds. The seeds were in controlled light (16/8-h light/dark cycle), luminance (30,000 lx), and temperature (22 ± 2°C) conditions. All of the experimental operations were aseptic. The germination index and eight root parameters were observed and determined.

### Determination of GA secretion.

The determination of GA secretion by B. altitudinis LZP02 and the strains treated with citric acid was measured under the same conditions, with shaking (59).

### Statistical analysis.

Analysis was conducted via SPSS 24.0 or GraphPad Prism 6.01. One-way ANOVA and *t* test were used to statistical analysis. The details are described in the figure legends.

### Data availability.

The raw sequence reads of the transcriptome have been deposited in NCBI under the accession number PRJNA849272.
