# Supplementary material for: Citric Acid in Rice Root Exudates Enhanced the Colonization and Plant Growth-Promoting Ability of Bacillus altitudinis LZP02
Source: Microbiol Spectr. 2022 Oct 20;10(6):e01002-22. doi: 10.1128/spectrum.01002-22 (PMC9769925; doi:10.1128/spectrum.01002-22)
Supplement: Supplemental file 1 — Tables S1-S6. Download spectrum.01002-22-s0001.pdf, PDF file, 0.1 MB [file spectrum.01002-22-s0001.pdf]

Supplementary Table S1. Information on genes from the RNA-Seq for bacterial chemotaxis pathway, and the associated with biofilm formation (N1)

Gene ID	Gene name	Log2(FC)	Regulate	Pathway	Gene description
KIV12_RS05340	*mcpA*	1.22516848656	up	Bacterial chemotaxis	methyl-accepting chemotaxis protein
KIV12_RS07150	*mcpA*	1.29456287595	up	Bacterial chemotaxis	methyl-accepting chemotaxis protein
KIV12_RS15080	*mcpC*	1.13132216293	up	Bacterial chemotaxis	methyl-accepting chemotaxis protein
KIV12_RS17945	*mcpA*	1.19723341027	up	Bacterial chemotaxis	methyl-accepting chemotaxis protein
KIV12_RS17120	*cheR*	1.33579233049	up	Bacterial chemotaxis	PAS domain-containing protein
KIV12_RS13780	*cheB*	1.05179218373	up	Bacterial chemotaxis	chemotaxis response regulator protein-glutamate methylesterase
KIV12_RS13775	*cheA*	1.00713131988	up	Bacterial chemotaxis	chemotaxis protein
KIV12_RS13885	*fliG*	1.02659783764	up	Bacterial chemotaxis	flagellar motor switch protein
KIV12_RS15200	*motB*	1.0288600958	up	Bacterial chemotaxis	flagellar motor protein
KIV12_RS05060	*rbsB*	1.12550714052	up	Bacterial chemotaxis	sugar-binding protein
KIV12_RS15260	*kinE*	1.34866630821	up	Two-component system	PAS domain-containing sensor histidine kinase
KIV12_RS15055	*kinA*	1.01835931283	up	Two-component system	PAS domain-containing sensor histidine kinase

Supplementary Table S2. Information on genes from cysteine and methionine metabolism, oxidative phosphorylation , pyrimidine metabolism pathway (N2)

Gene ID	Gene name	Log2(FC)	Regulate	Pathway	Gene description
PBLCHMIH _03126	*mtnK*	1.4732968 1181	up	Cysteine and methionine metabolism	Methylthioribose kinase
PBLCHMIH _03127	*mtnA*	1.5658432 1605	up	Cysteine and methionine metabolism	Methylthioribose-1-phosphate isomerase
PBLCHMIH _00176	*ldh_1*	2.1460159 9319	up	Cysteine and methionine metabolism	L-lactate dehydrogenase
PBLCHMIH _03122	*mtnW*	1.1972334 1027	up	Cysteine and methionine metabolism	2,3-diketo-5-methylthiopenty l-1- phosphate enolase
PBLCHMIH _03120	*mtnB*	1.4451759 2495	up	Cysteine and methionine metabolism	Methylthioribulose-1-phosph ate dehydratase
PBLCHMIH _03121	*mtnX*	1.7125914 5277	up	Cysteine and methionine metabolism	hydroxy-3-keto-5-methylthio pentenyl- 1-phosphate phosphatase
PBLCHMIH _00730	*metE_1*	1.0325645 1644	up	Cysteine and methionine metabolism	5-methyltetrahydropteroyltrig lutamate-- homocysteine methyltransferase
PBLCHMIH _01589	*metK*	1.3005455 7919	up	Cysteine and methionine metabolism	S-adenosylmethionine synthase
PBLCHMIH _03119	*mtnD*	1.0969787 0203	up	Cysteine and methionine metabolism	Acireductone dioxygenase
PBLCHMIH _01670	*msrC*	1.1340715 6315	up	Cysteine and methionine metabolism	Free methionine-R-sulfoxide reductase
PBLCHMIH _00908	*speE*	1.6245753 2388	up	Cysteine and methionine metabolism	Polyamine aminopropyltransferase
PBLCHMIH _00828	*qoxA*	2.0086388 1564	up	Oxidative phosphorylation	Quinol oxidase subunit 2
PBLCHMIH _00829	*qoxB*	1.6210473 7849	up	Oxidative phosphorylation	Quinol oxidase subunit 1
PBLCHMIH _00774	*cydA*	1.4211492 6574	up	Oxidative phosphorylation	Cytochrome bd ubiquinol oxidase subunit 1
PBLCHMIH _01376	*ndh*	1.4189120 3242	up	Oxidative phosphorylation	NADH dehydrogenase
PBLCHMIH _02991	*ctaA*	1.5132190 996	up	Oxidative phosphorylation	Heme A synthase
PBLCHMIH _00831	*qoxD*	1.4630968 8887	up	Oxidative phosphorylation	Quinol oxidase subunit 4
PBLCHMIH _00830	*qoxC*	1.2172600 229	up	Oxidative phosphorylation	Quinol oxidase subunit 3

PBLCHMIH_02735	*nrdE*	1.03569238773	up	Pyrimidine metabolism	Ribonucleoside-diphosphate reductase subunit alpha
PBLCHMIH_02912	*pyrE*	1.61302305744	up	Pyrimidine metabolism	Orotate phosphoribosyltransferase
PBLCHMIH_02918	*pyrC*	2.4298416488	up	Pyrimidine metabolism	Dihydroorotase
PBLCHMIH_02919	*pyrB*	4.50844441068	up	Pyrimidine metabolism	Aspartate carbamoyltransferase
PBLCHMIH_02026	*yqeN*	1.05873919709	up	Pyrimidine metabolism	putative protein
PBLCHMIH_00929	*pyrG*	1.57919324411	up	Pyrimidine metabolism	CTP synthase
PBLCHMIH_02923	*pyrR*	4.19779393912	up	Pyrimidine metabolism	Bifunctional protein
PBLCHMIH_02913	*pyrF*	1.15446426029	up	Pyrimidine metabolism	Orotidine 5'-phosphate decarboxylase

Supplementary Table S3. Information on genes from transcription (N2 COG functional annotation)

Gene ID	Gene name	Log2(FC)	*P*-adjust	Gene description
PBLCHMIH_03148	*tnrA*	1.34629169288	5.14305E-23	HTH-type transcriptional regulator
PBLCHMIH_00079	-	1.77681018609	2.11511E-45	hypothetical protein
PBLCHMIH_01769	*fadR*	1.03965402979	6.24903E-08	Fatty acid metabolism regulator protein
PBLCHMIH_00077	-	1.01551419926	8.83979E-06	hypothetical protein
PBLCHMIH_02132	*mntR*	1.30862864465	1.95840E-17	HTH-type transcriptional regulator
PBLCHMIH_03408	-	1.15777010322	1.23187E-05	hypothetical protein
PBLCHMIH_02874	*rnc*	1.27064948604	9.04869E-36	Ribonuclease 3
PBLCHMIH_01228	*opcR_1*	1.01877140085	2.41649E-18	HTH-type transcriptional repressor
PBLCHMIH_00403	*nusG*	1.09071091626	1.31138E-14	Transcription termination/antitermination protein
PBLCHMIH_01229	*opuCA*	1.63724252769	9.01868E-50	Glycine betaine/carnitine transport ATP-binding protein
PBLCHMIH_02263	*scpB*	1.57084286605	1.10574E-15	Segregation and condensation protein B
PBLCHMIH_01033	*csbC*	2.58912761156	1.45663E-16	putative metabolite transport protein
PBLCHMIH_00103	*abh_1*	1.78979638887	2.1731E-06	Putative transition state regulator
PBLCHMIH_00993	*slyA_2*	1.04987053733	5.68913E-08	Transcriptional regulator
PBLCHMIH_03165	*ohrR*	1.33759195087	4.35866E-12	Organic hydroperoxide resistance transcriptional regulator
PBLCHMIH_02033	*hrcA*	1.42443823948	9.45707E-22	Heat-inducible transcription repressor
PBLCHMIH_01230	*opuCB*	1.99222083586	1.25781E-30	Glycine betaine/carnitine transport system permease protein
PBLCHMIH_03902	-	1.01889287252	1.52001E-07	hypothetical protein

Supplementary Table S4. Information on genes from GO enrichment analysis (N2)

Gene ID	Gene name	Gene description	GO ID
PBLCHMIH_01589	*metK*	S-adenosylmethionine synthase	GO:0046500;
PBLCHMIH_03126	*mtnK*	Methylthioribose kinase	GO:0046500; GO:0019509
PBLCHMIH_03127	*mtnA*	Methylthioribose-1-phosphate isomerase	GO:0046500; GO:0019509
PBLCHMIH_03122	*mtnW*	2,3-diketo-5-methylthiopentyl-1-phosphate enolase	GO:0046500; GO:0019509
PBLCHMIH_03120	*mtnB*	Methylthioribulose-1-phosphate dehydratase	GO:0046500; GO:0019509
PBLCHMIH_03121	*mtnX*	2-hydroxy-3-keto-5-methylthiopentenyl-1-phosphate phosphatase	GO:0046500; GO:0019509
PBLCHMIH_03119	*mtnD*	Acireductone dioxygenase	GO:0046500; GO:0019509
PBLCHMIH_02794	*rpsO*	30S ribosomal protein S15	GO:0006412; GO:0005840; GO:0019843; GO:0003735
PBLCHMIH_02027	*rpsT*	30S ribosomal protein S20	GO:0006412; GO:0005840; GO:0019843; GO:0003735
PBLCHMIH_02863	*rplS*	50S ribosomal protein L19	GO:0006412;GO:0005840; GO:0003735
PBLCHMIH_02885	*rpmB*	50S ribosomal protein L28	GO:0006412;GO:0005840; GO:0003735
PBLCHMIH_01102	*yvyD*	Ribosome hibernation promotion factor	GO:0005840
PBLCHMIH_02037	*prmA*	Ribosomal protein L11 methyltransferase	GO:0005840
PBLCHMIH_00401	*rplA*	50S ribosomal protein L1	GO:0006412; GO:0005840; GO:0019843; GO:0003735
PBLCHMIH_00402	*rplK*	50S ribosomal protein L11	GO:0006412;GO:0005840; GO:0003735
PBLCHMIH_00353	*rplM*	50S ribosomal protein L13	GO:0006412;GO:0005840; GO:0003735
PBLCHMIH_00554	*rpsR*	30S ribosomal protein S18	GO:0006412; GO:0005840; GO:0019843; GO:0003735
PBLCHMIH_00552	*rpsF*	30S ribosomal protein S6	GO:0006412; GO:0005840; GO:0019843; GO:0003735
PBLCHMIH_01752	*rplT*	50S ribosomal protein L20	GO:0006412; GO:0005840; GO:0019843; GO:0003735
PBLCHMIH_01751	*rpmI*	50S ribosomal protein L35	GO:0006412;GO:0005840; GO:0003735
PBLCHMIH_00469	*ctc*	General stress protein CTC	GO:0006412;GO:0005840; GO:0003735
PBLCHMIH_00398	*rplL*	50S ribosomal protein L7/L12	GO:0006412;GO:0005840; GO:0003735

PBLCHMIH_00399	*rplJ*	50S ribosomal protein L10	GO:0006412;GO:0005840; GO:0003735
PBLCHMIH_01852	*rplU*	50S ribosomal protein L21	GO:0006412; GO:0005840; GO:0019843; GO:0003735
PBLCHMIH_01853	-	hypothetical protein	GO:0005840
PBLCHMIH_00776	*cydD*	ATP-binding/permease protein	GO:0015849; GO:0006865; GO:0046942
PBLCHMIH_03765	*opuE*	Osmoregulated proline transporter	GO:0015849; GO:0006865; GO:0046942
PBLCHMIH_00281	*yifK*	putative transport protein	GO:0015849; GO:0006865; GO:0046942
PBLCHMIH_00692	*gabP_1*	GABA permease	GO:0015849; GO:0006865; GO:0046942
PBLCHMIH_01251	*gltT_1*	Proton/sodium-glutamate symport protein	GO:0015849; GO:0006865; GO:0046942
PBLCHMIH_01680	*argO*	Arginine exporter protein	GO:0015849; GO:0006865; GO:0046942

Supplementary Table S5. qRT-PCR primer sequence information of N1

Gene		Primer sequence	Length
	16S rRNA-F	5' TGGAAACTGGGAAACTTGA 3'	
16s rRNA	16S rRNA-R	5' TTTACGGCGTGGACTACC 3'	180 bp
	05340-F	5' AATTCTGGAATCCATCCGTGAT 3'	
mcpA	05340-R	5' TTCAACAGCCGCCGACAT 3'	166 bp
	07150-F	5' ATTCCGAGATTCATAAAGG 3'	
mcpA	07150-R	5' TAGCGACAAACGCATAAC 3'	249 bp
	mcpC-F	5' ACGGATGAAGTGACCCAA 3'	
mcpC	mcpC-R	5' GACGAGCAGACCAAGAGC 3'	246 bp
	17945-F	5' ATCAAATAAGGGCGGTAA 3'	
mcpA	17945-R	5' GACGCATCTAAGTCAATCC 3'	228 bp
	cheR-F	5' ATGAGGAGTGCGAACCAA 3'	
cheR	cheR-R	5' CAATGATCGACGGAATGA 3'	175 bp
	cheR-F	5' CAAGTTTCTCCTAAGCCGTTCA 3'	
cheB	cheR-R	5' TAGCCAGCGATGCCGTAA 3'	229 bp
	cheA-F	5' ATGATGGCGGCGGGATTA 3'	
cheA	cheA-R	5' GAGCCTTGACCTTCTGTTGAGT 3'	247 bp
	fliG-F	5' CTAGAAAGGCTGACCCAG 3'	
fliG	fliG-R	5' AGATGTGCGATCCATTAA 3'	176 bp
	motB-F	5' GCAGAGCACCGAAGAAAC 3'	
motB	motB-R	5' CTCGCCCAGAATCAAAGA 3'	230 bp
	rbsB-F	5' CAAGCGTCAAGTGTATGTC 3'	
rbsB	rbsB-R	5' CGCCGACTCTTCTATTCC 3'	208 bp

Supplementary Table S6. qRT-PCR primer sequence information of N2

Gene		Primer sequence	Length
	16S rRNA-F	5' TGGAAACTGGGAAACTTGA 3'	
16s rRNA	16S rRNA-R	5' TTTACGGCGTGGACTACC 3'	180 bp
	metk-F	5' TGACCGAAGGACATCCAG 3'	
metk	metk-R	5' ACCGCTTACTAATACGAGACC 3'	134 bp
	cydD-F	5' GGCAACGCCTTATGTCTT 3'	
cydD	cydD-R	5' TTTCTCACGTCTGCTCCA 3'	117 bp
	qoxD-F	5' TCCTCAGCAGAGCACGAA 3'	
qoxD	qoxD-R	5' CAGACCCGATGACGAAAA 3'	268 bp
	qoxC-F	5' ATGCGGGATTGCCGTTCA 3'	
qoxC	qoxC-R	5' TCGCTGCTGTTTGTGGTGTT 3'	281 bp
	qoxA-F	5' CTTCTTGTTCAGAGCCATTA 3'	
qoxA	qoxA-R	5' AAGCGTATTCCCGTGTAG 3'	268 bp
	rpsO-F	5' TTCAGATCGCTGTCCT 3'	
rpsO	rpsO-R	5' CGTAAGCCTAGTTTGT 3'	184 bp
	flgG-F	5' ACCGTTTCTGTTGTTGAG 3'	
flgG	flgG-R	5' CTGAGTGTTTGGCGATAT 3'	119 bp
	fliM-F	5' TCGGTTGACCAAGTGCCA 3'	
fliM	fliM-R	5' CGCCCATCAGCCTATCCA 3'	151 bp
